# Sci-tech arts on Chang’e-5 lunar soil

**DOI:** 10.1016/j.xinn.2022.100300

**Published:** 2022-08-11

**Authors:** Wei Yang, Yi Wang, Lin Gao, Di Zhang, Jie Yang, Zhijie Qiu

**Affiliations:** 1 Key Laboratory of Earth and Planetary Physics, Institute of Geology and Geophysics, Chinese Academy of Sciences, Beijing 100029, China; 2 Visual Art Innovation Institute, Central Academy of Fine Arts, Beijing 100102, China; 3 Beijing Key Laboratory of Mobile Computing and Pervasive Device, Institute of Computing Technology, Chinese Academy of Sciences, Beijing 100190, China

Since ancient times, humans have created artistic works using state-of-the-art techniques, thus exploring the relationship between technological advances and human developments. These activities are the prototype of “sci-tech art.” For example, the precise control of smelting and casting in the Bronze Age gave birth to the *Chime* bells, the *Zun* wine vessel, and the *Jue* wine cup. The integration of material and firing technology resulted in ceramic art, which became one of the symbols of China. The rise of the study of anatomy reshaped how people saw the human body and the structure of the world around them, ultimately promoting the Renaissance. The development of photography technology led to the seventh art of films and cinema, which is now being reformed by the latest computer-graphics technologies. There is no doubt that new technologies will continue to move art forward and build a better world in the future.^1^

Conversely, the vision of art also leads to the development of science and technology. For example, the artistic imagination of the starry sky has always been the driving force for deep-space exploration. *De la Terre à la Lune* by Jules Verne inspired the first pioneers of astronautics to dedicate their lives to solving the problems of space flight. Finally, in 1969, the Apollo 11 mission achieved the human dream of landing on the Moon. During the next 7 years, the six Apollo and three Luna missions returned approximately 382 kg of lunar samples, which have significantly enhanced our understanding of the formation and evolution of the Moon.

The lunar samples were all collected from a regolith layer that nearly covers the entire surface of the Moon. It is the actual boundary layer between the solid Moon and space, providing critical information about both the Moon and the space environment around it.^2^ Besides some rocks, many samples are fine-grained lunar soil with particles <1 cm in size. Although scientists have learned much about the physical properties and mineral and chemical compositions of the lunar soil,^2^ the general public know very little about it. The main reason is that microscopy and image processing technologies from 40 years ago could not present such fine lunar soil particles to the public. The artistic presentation of these particles could not be created, and consequently, the dissemination of the current knowledge was hindered, which in turn prevented further innovations in science, technology, and artistic presentation.

In 2020, Chang’e-5, China’s first lunar sample return mission, brought back 1.731 kg of lunar samples from the northeastern Oceanus Procellarum,^3^ making China the third country to return samples from the Moon after the United States and the Soviet Union. These new lunar samples have attracted widespread attention and ignited a research bonanza in China.^4^ Now, times have changed, and significant advances have been achieved in microscopy and image processing technologies over the past 40 years. These state-of-the-art techniques, such as micrography, scanning electron microscopy, three-dimensional (3D) X-ray microscopy, multi-source data fusion, geometric modeling and texture synthesis, and high-quality 3D rendering, not only help with more scientific discoveries but also provide better presentations to the public. After integrating the current understanding of the Moon’s surface and the history of human lunar exploration, these presentations can provide a wider artistic vision, leading to more exciting and interesting dissemination of information and stimulating further enthusiasm for deep-space exploration.

The Chang’e-5 lunar soil sample studied here was from one scooped sample (CE5C0600YJFM00402, ∼1.5 g) allocated by the China National Space Administration. Artists from the Central Academy of Fine Arts participated in the research, from the acquisition of multiple microscopy data to the reconstruction of 3D models.

**Figure 1:**
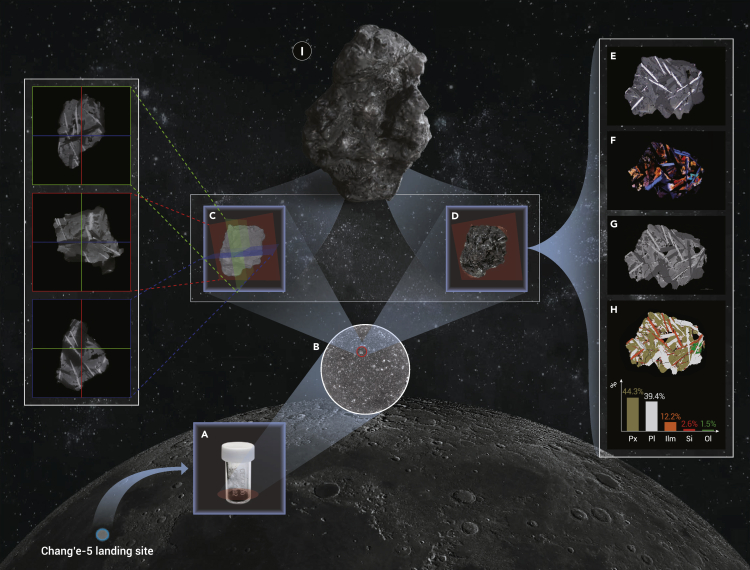
An example of the Chang’e-5 lunar soil particle (CE5C0600YJFM00402, 137) (A) The Chang’e-5 lunar soil sample CE5C0600YJFM00402 (1500 mg). (B) A cross-section of the soil sample in the quartz vial from the X-ray microscope. (C) 3D tomography and internal structure of the particle acquired by the X-ray microscope. (D) Focus stacking micrograph of the particle, showing the position where the thin section was prepared. (E) The reflected light image of the section. (F) The crossed polarized light image of the section. (G) The backscatter electron image of the section. (H) The mineral mapping image of the section. Px, pyroxene; Pl, plagioclase; Ilm, ilmenite; Si, silica; Ol, olivine. (I) The 3D visualization of this particle reconstructed based on the 3D tomography and focus stacking micrograph. All data and models are available at http://the-innovation.org/online-contents/sci-tech-art-lunar-soil.

We scanned the 1.5 g sample in a quartz vial (Figure 1A) using an FEI Heliscan X-ray microscope for 3D tomography at a voxel size of about 10 μm (Figure 1B). Then, the sample was sieved by a 355 μm stainless steel sieve. The >355 μm fraction contains 146 particles. Each particle was scanned using the same microscope at a higher resolution of about 1 μm. Based on the internal structures revealed by the X-ray microscope, the different types of particles can be identified, including 40 basalt, 36 breccia, 37 agglutinate, 32 glass, and 1 olivine fragments. We took focus stacking micrographs for each particle and selected 42 particles to prepare sample mounts. Backscattered electron images and elemental mapping were carried out on a Thermo Scientific Apreo scanning electron microscope. Then, we chose 13 particles to prepare thin sections 30 μm thick and took reflected light and crossed polarized light micrographs. On the basis of the above 2D and 3D image data, we performed the 3D reconstruction and multi-source data fusion of the lunar soil particles.

Figure 1 exhibits an example of one lunar soil particle (CE5C0600YJFM00402, 137), which is approximately 700×600×400 μm in size. The X-ray microscope revealed its internal structure (Figure 1C), suggesting it is a basalt fragment. Its focus stacking micrography (Figure 1D) shows that it mainly consists of pyroxene (brown), plagioclase (white), and ilmenite (dark). Based on the reflected light, crossed polarized light, backscatter electron, and mineral distribution images of one cross-section (Figures 1E–1H), this basalt fragment shows a subophitic texture, where pyroxene partially encloses the plagioclase. It contains 44.3% pyroxene, 39.4% plagioclase, 1.5% olivine, 12.2% ilmenite, and 2.6% silica. This basalt indicates that volcanic activity once occurred on the Moon, and the small grain size of the fragment reflects the continuous space weathering on the lunar surface. Based on the 3D topography and the focus stacking micrograph, the 3D visualization of this particle was reconstructed (Figure 1I), taking into account the intensity and incidence angle of the sunlight at the Chang’e-5 landing site. Thus, an amazing scientific and yet artistic way to present the fine lunar soil particles to the public was created.

In this study, artists have been involved from the beginning of the scientific research, giving an artistic vision for image acquisition and 3D model reconstruction. This collaboration opens up a new paradigm of fusion of science and art. This way, artistic creations can bring scientific discoveries to the public, presenting details of the research and making science more popular. Shortly, artists will create artistic works based on the current results, making science more aesthetic. With the development of microscopy, image processing, and virtual and augmented reality technologies, this new paradigm will be widely applied in other fields of scientific research, promoting the deep integration of science, technology and art.
